# Anemia Due to Unexpected Zinc-Induced Copper Deficiency

**DOI:** 10.3390/hematolrep17040035

**Published:** 2025-07-17

**Authors:** Nicholas Chun, Shehla Aman, Dan Xu, Jun Wang, Craig Zuppan, Albert Kheradpour

**Affiliations:** 1 Department of Pathology and Laboratory Medicine, Loma Linda University Medical Center, Loma Linda, CA 92354, USA; saman@llu.edu (S.A.); dxu@llu.edu (D.X.); jwang@llu.edu (J.W.); czuppan@llu.edu (C.Z.); 2 Division of Pediatric Hematology/Oncology, Loma Linda University Children’s Hospital, Loma Linda, CA 92354, USA; akheradp@llu.edu

**Keywords:** anemia, leukopenia, neutropenia, sideroblast, zinc, copper, sideroblast, dietary

## Abstract

Anemia due to acquired copper deficiency is most commonly the result of malabsorption or dietary deficiency. However, it can occasionally be due to excess zinc intake, which impairs the absorption of copper. Copper deficiency may result in vacuolated erythroid and myeloid precursors in the bone marrow, and sometimes features resembling myelodysplasia that, although not specific, may be an important clue to the diagnosis. Background and Clinical Significance: We report bone marrow findings in a child with anemia due to zinc-induced copper deficiency. Case Presentation: An 18-year-old female with cerebral palsy admitted for respiratory failure was found to have anemia and leukopenia with absolute neutropenia. A bone marrow smear showed occasional ring sideroblasts. Additional testing revealed reduced serum copper and elevated serum zinc. Further inquiry uncovered a several-year history of high-dose zinc supplementation. Conclusions: It is important to consider copper deficiency as a potential etiology in patients with anemia and neutropenia, as it may otherwise be mistaken for vitamin B12 deficiency or myelodysplasia. The presence of small vacuoles in hematopoietic precursors is an important clue to the diagnosis and may help avoid ineffective interventions.

## 1. Introduction and Clinical Significance

Copper deficiency is a rare but well-documented cause of anemia, neutropenia, and sideroblastic anemia [1]. Therefore, it is important to recognize copper deficiency as a potential etiology in patients presenting with cytopenias. Copper deficiency can be due to a variety of reasons, including dietary deficiency, malabsorption, excess excretion, Wilson disease, Menkes syndrome, and excess ingestion of zinc in the form of supplements, dental fixatives, and zinc pennies [2,3,4]. Copper deficiency can lead to reversible hematological and irreversible neurological manifestations [5,6]. However, due to its low prevalence and nonspecific hematological and clinical manifestations, copper deficiency is often misdiagnosed as myelodysplastic syndrome due to similar hematopathological findings [7]. The bone marrow findings of copper deficiency include hypocellularity, vacuolization of erythroid and myeloid precursors, and ring sideroblasts [8,9]. Earlier detection of copper deficiency can avoid future morbidity of neurological deficits, cytopenias, and continued ineffective interventions. In addition, it is important to consider a rare cause of copper deficiency that has been reported more in the elderly population, zinc supplementation [10]. We describe the case of a young adult patient with a complex medical history in whom copper deficiency led to anemia and neutropenia due to long-term supplemental use of high-dose zinc.

## 2. Case Presentation

### Lennox–Gastaut Syndrome

The patient is an 18-year-old female with history of hypoxic–ischemic encephalopathy (HIE) due to non-accidental trauma (NAT), intellectual disability, cerebral palsy (CP), dysphagia with gastrostomy tube (GT), gastroesophageal reflux (GER) status post Nissen fundoplication, Lennox–Gastaut Syndrome (LGS) status post vagus nerve stimulation (VNS) device placement, scoliosis status post spinal fusion, restrictive lung disease, tracheostomy with ventilator dependence, and history of recurrent pneumonias brought to the emergency department (ED) for hypoxia. A few days before presenting to the ED, the patient’s mother reported that her daughter had a seizure that lasted for 20 min that required Diazepam and later noted foul smelling stools, tachycardia, abdominal distension, and increased paleness.

**Figure 1:**
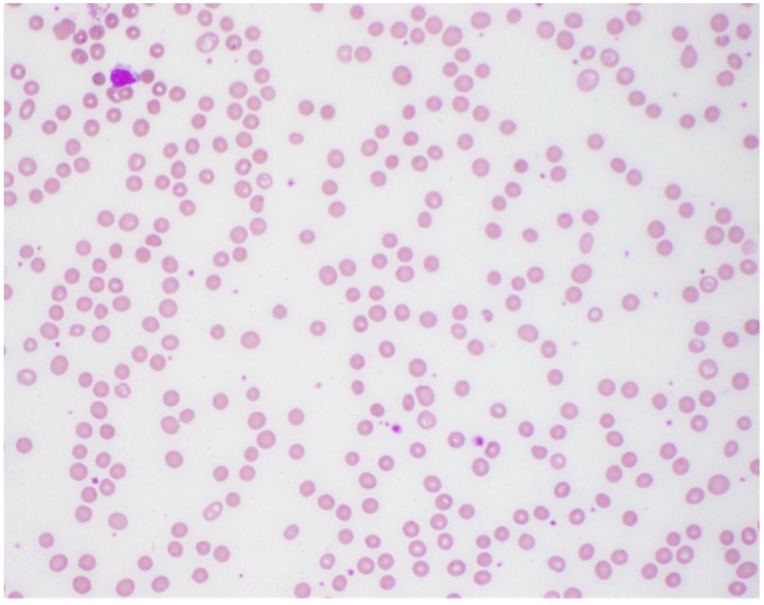
Peripheral smear showing moderate normocytic normochromic anemia.

The patient was admitted for respiratory failure. As the etiology for the respiratory failure was being worked up, the initial complete blood count (CBC) with differential reported significant findings of moderate, borderline normocytic (MCV: 96.1 fL) anemia and marked leukopenia with neutropenia, based on the hospital system’s determined reference ranges (Table 1). To rule out a secondary cause of the anemia, tests assessing the levels of bilirubin, vitamin B12, and folic acid were ordered, which were all within normal ranges. The peripheral smear showed moderate normocytic normochromic anemia as shown in Figure 1. After medication reconciliation, it was noted that the patient had a several-year history of Felbamate use to help manage her seizures. While pancytopenia is a known side effect of Felbamate, it did not adequately explain the patient’s newfound anemia as there was no prior abnormal blood cell counts since starting the medication. At this time, a new acute process was suggested with differentials including febrile neutropenia, viral marrow suppression, antiepileptic drug (AED) marrow suppression, and myelodysplastic syndrome (MDS).

**Figure 2:**
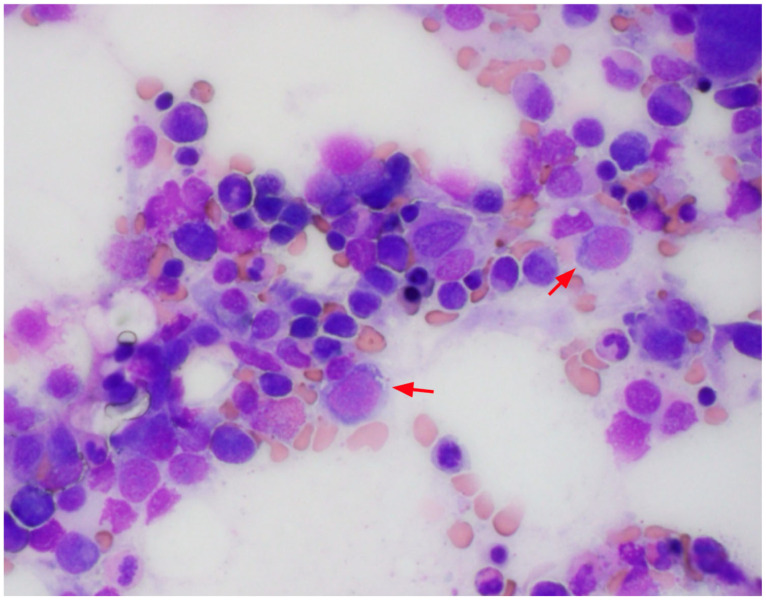
Bone marrow aspirates with cytoplasmic vacuoles seen in erythroid precursors (red arrows).

**Figure 3:**
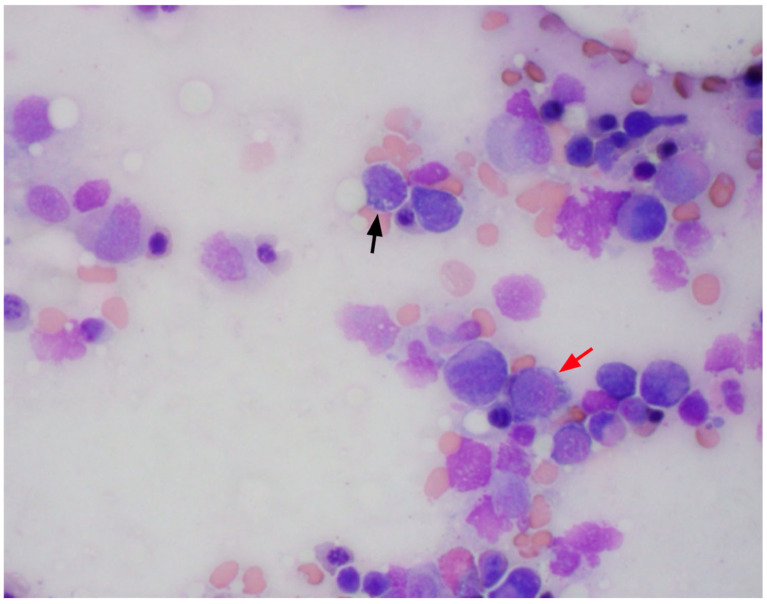
Bone marrow aspirates with cytoplasmic vacuoles seen in erythroid (red arrow) and myeloid precursors (black arrow).

A bone marrow biopsy was performed, and the marrow was revealed to be hypocellular for her age (60–70%). There was no histologic evidence of marrow involvement by leukemia, lymphoma, or metastatic malignancy. The bone marrow smears showed multiple small cytoplasmic vacuoles in some of the erythroid precursors and myeloid precursors as shown in Figure 2 and Figure 3, as well as occasional ring sideroblasts (<10%). These findings were recognized as possible signs of copper deficiency and prompted secondary testing for serum copper and zinc. As these serum tests were not available in the hospital laboratory, send-out tests were ordered, and a peripheral blood sample was sent for analysis. Serum copper was markedly decreased (<0.10 mcg/mL) and serum zinc was elevated (1.80 mcg/mL) (Table 2).

Further inquiry with the patient’s mother revealed the use of high-dose zinc supplementation for the past three years while on a ketogenic diet as directed by a nutritionist from an outside hospital system. The care team counseled the patient’s family on the effects of zinc supplementation and discontinued its use. The patient was started on IV copper chloride supplementation for 5 days with gradual improvement of the patient’s white blood count and absolute neutrophil count. The chromosomal analysis of the marrow resulted as overall normal. The remainder of the patient’s hospital stay was focused on managing her respiratory symptoms and seizures which were complicated by fever. On admission, the patient was started on antibiotics for concern for pneumonia. However, chest X-rays did not appear to show signs of pneumonia. Sputum trachea aspirate culture was positive with moderate growth of Gram-negative rods, suspicious for *Pseudomonas aeruginosa*. The patient was then administered the appropriate antibiotics based on the bacterial sensitivities report with improvement of her respiratory symptoms. After it was found that the likely cause of anemia was copper deficiency, the neurologists restarted the patient’s home AED regimen, with improvement of seizure activity. On several occasions the patient’s hemoglobin (Hgb) levels dropped below 7 g/dL, in which she was transfused one unit of red blood cells each time. Upon resolution of her marked anemia, the patient was discharged.

Approximately one month after cessation of zinc supplementation, the patient presented for follow-up. After the 5-day course of IV copper chloride, the patient was prescribed home supplementation of 1 mg copper chloride, until a serum copper level was re-checked. A follow-up CBC and serum copper level was ordered to monitor the patient’s anemia, which reported no abnormalities (Table 1 and Table 2). At this time, the copper supplementation was discontinued. An additional serum zinc level was not ordered by the clinician at this time, as the anemia and copper levels had resolved.

## 3. Discussion

This case underscores the intricate interactions between copper and zinc, unraveling not only the complexities of micronutrient metabolism but also the diagnostic challenges entwined with differentials.

Zinc, predominantly absorbed in the duodenum and jejunum [11], binds to metallothionein—a primarily intracellular protein regulating various metals, including copper. Excessive zinc stimulates enterocytes to produce more metallothionein, thereby decreasing free zinc concentrations. However, due to copper’s higher affinity to metallothionein than zinc, this leads to a decrease in serum copper levels [12]. Additionally, copper, a crucial cofactor for enzymes in heme biosynthesis, plays a pivotal role in facilitating the insertion of iron into protoporphyrin IX, a process catalyzed by ferrochelatase. Copper deficiency can impede this crucial step in hemoglobin production [13]. Moreover, copper influences intestinal iron absorption through the alteration of DNA-binding proteins and mitochondrial iron homeostasis, contributing to factors that may account for anemia [10,14].

The bone marrow findings, presence of ringed sideroblasts and vacuolated erythroid and myeloid precursors, prompted a clinical investigation into copper and zinc levels. The vacuolated erythroid precursors highlight the impact of copper deficiency on heme synthesis, underscoring the non-specific yet pivotal role of bone marrow examination in guiding the diagnostic process. Based on these findings and the additional copper and zinc serum levels, prompt correction led to the resolution of anemia and leukopenia with neutropenia. This case serves as a testament to the reversible nature of this condition when identified early.

As discussed previously, to determine zinc toxicity, copper levels must be assessed as well. Clinically, copper deficiency can be very difficult to assess, with some cases taking months to years to diagnose. The common manifestations include leukopenia, anemia (microcytic, normocytic, or macrocytic), myelopathy, and peripheral neuropathy. Of note, one pertinent negative lab finding that has been reported in these patients is normal platelet counts [1,2,3,5,7], though this finding requires further studies to confirm significance. Identifying chronic zinc ingestion as the cause is crucial to prevent irreversible consequences, such as ascending sensorimotor polyneuropathy syndrome, especially in patients with preexisting neurological impairment.

Beyond copper deficiency, vacuolated erythroid and myeloid precursors can be associated with various conditions, including Pearson syndrome [15], myelodysplastic syndromes [9], acute alcoholic intoxication [16], and Menkes disease [17]. However, several important negative findings collectively ruled these differential diagnoses less likely: the uneventful chromosomal analysis result and the absence of dysplastic features in the bone marrow; the lack of significant past medical history, laboratory findings, and family history related to these diagnoses.

## 4. Conclusions

We report a rare case of zinc-induced copper deficiency causing anemia and discuss its pathophysiology. This case emphasizes the importance of utilizing histologic features and understanding cellular and molecular mechanisms to connect the dots in a clinical puzzle. Copper’s role in hemoglobin synthesis as well as zinc’s regulatory effect contributes to the broader literature on the intricate interplay between zinc and copper metabolism. There are very few reports of zinc-induced copper deficiency, with none reporting on young adult patients or patients with multiple comorbidities that includes GT dependence. In addition, this case highlights the importance of a thorough history and physical exam and chart review.

Based on the rapid recovery of this patient’s neutropenia, IV copper chloride supplementation may be considered in the management of zinc-induced copper deficiency. As this patient had dysphagia and was GT-dependent, we did not explore oral copper supplementation; however, there is one report of an elderly patient that demonstrated sufficient treatment with oral copper gluconate over several months [10,18]. Consultation with a nutritionist and pediatric oncologist are essential for managing patients with multiple comorbidities and critical presentations.

Healthcare professionals, armed with a nuanced understanding of these mechanisms, can navigate the delicate balance of micronutrients for optimal patient care. As we unravel the complexities of zinc-induced copper deficiency anemia, we gain insights not only into pathophysiology but also elevate our awareness of the significance of early intervention in preventing potential complications.

## Figures and Tables

**Table 1:** CBC with differentials taken in the emergency department (Initial presentation), when copper supplementation was initiated (After two days IV copper chloride), and approximately one month after zinc supplement cessation (One month Post-treatment). The post-treatment CBC demonstrates resolution of the patient’s anemia and neutropenia.

CBC with Differential Reference Range and Units	Initial Presentation	After Two Days of IV Copper Chloride	One Month Post-Treatment
WBC 4.80–11.80 bil/L	1.69 Low	1.87 Low	5.08
RBC 3.60–5.30 tril/L	2.06 Low	2.60 Low	3.71
Hgb 10.5–15.0 g/dL	6.2 Low	7.8 Low	11.4
Hct 32.0–46.0%	19.8 Low	23.1 Low	34.7
MCV 77.0–96.0 fL	96.1	88.8	93.5
MCH 24.5–32.0 pg	30.1	30.0	30.7
MCHC 30.0–35.0 g/dL	31.3	33.8	32.9
RDW 12.0–15.0%	16.0 High	16.2 High	14.6
Plts 130–460 bil/L	246	278	293
MPV 0.0–15.0 fL	10.2	9.8	9.7
ANC 1.5–8.3 bil/L	0.4 Low	1.3 Low	3.3
Segs, Manual 38–79%	19 Low	64	65
Bands, Manual 0–7%	3	4	--
Lymphs, Manual 7–40%	67 High	20	30
Monos, Manual 3–10%	5	6	2
Eosins, Manual 0–6%	3	4	2
Basophils, Manual 0–1%	--	--	1
Variant Lymphs 0–3%	2	1	--
Metamyelocyte %	1	--	--

**Table 2:** Serum zinc and copper level tests. The patient’s zinc levels were taken due to suspicion of zinc toxicity (pre-treatment). The serum copper level test reported an abnormal (low) result, taken due to suspicion of zinc-induced copper deficiency (pre-treatment) and taken approximately one month after zinc supplement cessation (post-treatment).

Serum Copper Levels Reference Range and Units	Initial Presentation	One Month Post-Treatment
Zinc (mcg/mL) 0.66–1.10 mcg/mL	1.37 High	
Copper (mcg/mL) 0.75–1.45 mcg/mL	<0.10 Low	1.00

## Data Availability

No new data was created in this case report.

## Abbreviations

The following abbreviations are used in this manuscript:

g	Grams
µg/mcg	Microgram
dL	Deciliters
mL	Milliliters
CBC	Complete Blood Count
WBC	White blood count
RBC	Red blood cells
Hgb	Hemoglobin
Hct	Hematocrit
MCV	Mean corpuscular volume
MCH	Mean corpuscular hemoglobin
MCHC	Mean corpuscular hemoglobin concentration
RDW	Red cell distribution width
Plts	Platelets
MPV	Mean platelet volume
